# A Highly Sensitive BRET-Based Reporter for Live-Cell Detection of HIV-1 Protease Activity and Inhibitor Screening

**DOI:** 10.3390/v17101391

**Published:** 2025-10-19

**Authors:** Matteo Centazzo, Atalie Verra-Victoria Djossou, Silvia Pavan, Gualtiero Alvisi

**Affiliations:** Department of Molecular Medicine, University of Padua, 35121 Padova, Italy; centazzo@ingm.org (M.C.); djossouatalie@gmail.com (A.V.-V.D.); silvia.pavan.1@unipd.it (S.P.)

**Keywords:** HIV-1, BRET, protease inhibitors, reporter, drug screening, NanoLuc, mNeonGreen, live-cell assay, high-throughput screening, antiviral drug discovery

## Abstract

Given their role in viral polyprotein processing, viral proteases (PRs) are excellent targets for antiviral therapy. Most assays developed for screening PR inhibitors are in vitro assays, and therefore have several limitations, including the inability to account for cell permeability, toxicity and the need for compounds activation within cells. The development of cellular reporters overcoming these limitations is therefore highly desirable. In this study, we developed two different Bioluminescence Resonance Energy Transfer (BRET)-based reporters for Human Immunodeficiency virus-1 (HIV-1) PR, allowing the simultaneous monitoring of cell viability and HIV-1 PR activity. The reporters employ two different BRET pairs as donor and acceptor moieties: Renilla luciferase (RLuc) with Yellow Fluorescent Protein (YFP), and Nano luciferase (NLuc) with mNeonGreen (mNG), both linked by the HIV-1 p2/p7 cleavage site. While both reporters specifically detected HIV-1 protease activity, mNG-p2/p7-NLuc exhibited higher sensitivity, increased energy transfer and better spectral separation between donor and acceptor emissions, resulting in a significantly higher BRET ratio. mNG-p2/p7-NLuc was used to quantify the effect of a panel of protease inhibitors in living cells, assessing simultaneously cell viability and HIV-1 PR activity. Additionally, it was employed to measure the potency of well-known HIV-1 PR inhibitors. Together, these findings demonstrate the utility of the mNG-p2/p7-NLuc reporter as a cell-based tool for the evaluation of HIV-1 PR activity and inhibitor efficacy. Its dual-readout capability provides a valuable platform for antiviral drug screening in physiologically relevant conditions.

## 1. Introduction

Viral proteases (PRs) are encoded by members of several viral families and represent major targets for antiviral therapy due to their crucial role in processing virus-encoded polyproteins in their mature components and degrading host cell proteins [1,2]. The importance of active and well-tolerated antivirals is exemplified by the significant positive impact on human health following the approval of highly active PR inhibitors against human immunodeficiency virus type 1 (HIV-1), anti-hepatitis C virus (HCV), and severe acute respiratory syndrome coronavirus 2 (SARS-CoV-2) which are used in combination therapy [3,4,5]. Unfortunately, current assays for the discovery of viral PR inhibitors are mostly performed in vitro and therefore have several limitations, including the inability to account for cell permeability and toxicity, as well as the failure to identify compounds that require cellular activation [6,7]. Several cell-based assays that rely on the simultaneous expression of the viral protease of interest and a reporter that produces a specific signal upon cleavage have been developed to address such limitations. These assays enable the evaluation of protease activity in the presence or absence of candidate inhibitors, with significant implications for high-throughput drug discovery and rapid quantification of viral infections. Nonetheless, they also present certain drawbacks, including low signal-to-noise ratios, limited sensitivity to protease inhibitors, and complexity of data analysis [8,9,10]. Among the different strategies used to monitor viral protease activity in living cells, bioluminescent resonance energy transfer (BRET) has emerged as particularly promising. Due to its versatility, BRET has been successfully applied in screening campaigns to identify inhibitors of protein–protein interactions [11]. A BRET-based HIV-1 protease (PR) reporter for use in living cells has been recently described; it relies on the expression of GFP2 and a Renilla luciferase (RLuc) flanking the HIV-1 p2/p7 cleavage site [12]. The use of RLuc as BRET donor in combination with either GFP2 or YFP presents some drawbacks, including the low signal-to-noise following substrate addition and significant spectral overlap between donor and acceptor emissions, which results in suboptimal performance. However, the recent development of new bioluminescent and fluorescent proteins has opened new possibilities for overcoming these issues, by enabling the use of more efficient BRET pairs. For example, Nanoluc luciferase (NLuc)—a luminescent protein engineered from the luciferase of a luminous deep-sea shrimp, *Oplophorus gracilirostris* [13]—offers several advantages over RLuc when used as a BRET donor [13]. First, its smaller size compared to RLuc (19.1 kDa vs. 36 kDa) makes it less likely to interfere with protein function, localization, or conformation of fusion proteins. Second, oxidation of its specific substrate furimazine generates an exceptionally bright bioluminescent signal, allowing detection of very low protein levels. In addition, oxidation of furimazine produces an emission peak at shorter wavelengths (460 nm) compared to coelenterazine oxidation by RLuc (481 nm), resulting in reduced spectral overlap between donor and acceptor emission. This significantly reduces background and increases BRET ratios using standard luminescence filters [14]. Furthermore, NLuc can also oxidize RLuc substrates such as coelenterazine and h-coelenterazine (h-CTZ), allowing a direct comparison of the two luciferases [15]. On the other hand, mNeonGreen (mNG) is a 26.6 kDa monomeric green-yellow, derived from *Branchiostoma lanceolatum*, which matures rapidly and exhibits excitation and emission peaks at 506 and 517 nm, respectively [16], making it an ideal BRET fluorescent acceptor for NLuc. Notably, the emission peak separation between NLuc and mNG is 57 nm, compared to only 46 nm for RLuc and YFP, which has excitation and emission peaks at 513 and 527 nm, respectively. Furthermore, mNG is 1.8 times brighter and has a higher quantum yield (0.80 vs. 0.61) than EYFP, further emphasizing its superiority as a BRET acceptor [17]. We report here the development of a novel BRET reporter for the detection of Human Immunodeficiency Virus 1 (HIV-1) PR activity in living cells, whereby the donor (NLuc) and the acceptor (mNG) are linked by a peptide linker endowed with the HIV-1 p2/p7 cleavage site. We demonstrate that the mNG-NLuc BRET pair exhibits lower background signal, higher BRET ratio, and superior sensitivity compared to the conventional YFP-RLuc pair after addition of h-CTZ [18,19,20]. Co-expression of HIV-1 PR, but not of a catalytically impaired mutant, resulted in dose-dependent reduction in the BRET ratio in cells expressing the mNG-p2/p7-NLuc reporter. In contrast, no reduction was observed with a control reporter lacking the p2/p7 cleavage site. Detection of fluorescent and bioluminescent signals allowed parallel quantification of both cell viability and PR activity in cells treated with a panel of protease inhibitors. We demonstrated that HIV-1 PR activity is effectively inhibited at micromolar concentrations by clinically approved inhibitors such as lopinavir, ritonavir, saquinavir and nelfinavir, whereas no inhibition was observed with compounds targeting unrelated viral proteases, including rupintrivir (Rhinovirus 3CL), simeprevir (Hepatitis C Virus NS3/4A), tannic acid (SARS-CoV-2 nsp5), and tolcapone (ZIKA NS3/4A).

## 2. Materials and Methods

### 2.1. Plasmids. Mammalian Expression Plasmids

YFP-p2/p7-RLuc and mNG-p2/p7-NLuc were synthesized by VectorBuilder (Neu-Isenburg, Germany). The construct pDESTnYFP-RLuc was generated by LR recombination using Gateway^TM^ technology (Thermo Fisher Scientific, Waltham, MA, USA), with pDNR207-RLuc as the entry clone and pDESTntYFP as the destination vector [21], as previously described [22,23]. The mNG-NLuc plasmid was generated by excising the p2/p7 encoding fragment from mNG-p2/p7-NLuc via BamHI and BglII (Promega, Madison, WI, USA) restriction digestion, followed by ligation of plasmid backbone. The RLuc-UL44 plasmid was previously described [18], while plasmid pcDNA3.1-NLuc-UL44 was generated by Gateway^TM^ LR recombination between pDNR207-UL44 [24] and pcDNA3.1-Nanoluc-GS-ccdB (Addgene #87070, kindly provided by Mikko Taipale, University of Toronto, Toronto, ON, Canada). Plasmids pcDNA4/TO-PR, and pcDNA4/TO-PR KO, encoding 2xStrepTagII-TEV-3xFLAG C-terminally tagged versions of either HIV-1 PR or its catalytically impaired D25N mutant [25] were generously supplied by Nevan J. Krogan (University of California, San Francisco, CA, USA).

### 2.2. Protease Inhibitors. Saquinavir Mesylate

Saquinavir (SAQ; Sigma-Aldrich, St. Louis, MO, USA, #S8451), rupintrivir (RUP; Sigma-Aldrich, #PZ0315), tolcapone (TOL; Sigma-Aldrich, #SML0150), tannic acid (TAN; Sigma-Aldrich, #1007731000), nelfinavir mesylate (NEL; Selleckchem, Houston, TX, USA, #S4282) and simeprevir (SIM; Selleckchem, #S5015) were dissolved in DMSO to prepare 10 mM stock solutions. Lopinavir (LOP; Selleckchem, #S1380) and ritonavir (RIT; Selleckchem #S1185) were purchased as 10 mM stocks in DMSO (Sigma-Aldrich, #D4540). All stock solutions were stored at −80 °C for long-term use, whereas single-use working aliquots were stored at −20 °C.

### 2.3. Cell Culture

HEK293T cells were maintained in Dulbecco’s Modified Eagle’s Medium (DMEM) supplemented with 10% (*v*/*v*) fetal bovine serum (FBS), 50 U/mL penicillin, 50 U/mL streptomycin, and 2 mM L-glutamine. Cells were passaged upon reaching confluence as previously described [26].

### 2.4. Western Blotting Analysis

HEK293T cells were seeded in 6-well plates (5 × 10^5^ cells/well). The following day, cells were either mock-transfected or transfected with appropriate amounts of the desired plasmid using Lipofectamine 2000 (Thermo Fisher Scientific, #11668027) as described in [27]. Specifically, 500 ng of BRET reporter encoding plasmid, was used per well, and when required, either 500 or 1000 ng of plasmid expressing HIV-1 PR was co-transfected. Six hours post-transfection (p.t.), the medium was replaced with either fresh DMEM or DMEM containing the HIV-1 PR inhibitor LOP (10 μM). At twenty-four hours p.t., cells were washed with ice-cold PBS and lysed in 150 μL of RIPA buffer supplemented with protease inhibitors (50 mM Tris-HCl, pH 7.4; 150 mM NaCl; 1% Triton X-100 (*v*/*v*); 1% sodium deoxycholate; 0.1% SDS; 1 mM EDTA, 17.4 μg/mL phenylmethylsulfonyl fluoride; 2 μg/mL aprotinin, and 4 μg/mL leupeptin) as described previously [28]. Protein concentrations were determined using the Micro BCA Protein Kit assay (Thermo Fisher Scientific, #23235) as previously described [29]. Equal amounts of protein (50 µg) from each sample were resolved by SDS-PAGE and analyzed by Western blotting as described previously [30]. The following primary mouse monoclonal antibodies were used: α-RLuc (Merck Millipore, Burlington, MA, USA, #MAB4400; 1:2000); α-NLuc (Promega, #MAB100261; 1:2000); α-tubulin (Sigma-Aldrich, #T6074; 1:8000); α-HIV-1 PR (Thermo Fisher Scientific, #1696; 1:2000). Membranes were incubated with goat a-mouse secondary antibody conjugated to horseradish peroxidase (Santa Cruz Biotechnology, Dallas, TX, USA, #sc-2055; 1:10,000). Chemiluminescent signals were acquired using the Alliance Mini imaging system (Uvitech, Cambridge, UK) and quantified using Fiji (NIH, Bethesda, MD, USA).

### 2.5. Spectral Measurements

To measure spectral properties of bioluminescent and fluorescent proteins in living cells, HEK293T cells were seeded in twenty-four-well plates (1 × 10^5^ cells/well). The following day, cells were transfected using Lipofectamine 2000 (Thermo Fisher Scientific, #11668027,) according to the manufacturer’s recommendations. Transfections included 150 ng of plasmids encoding either HIV-1 PR BRET reporters (YFP-p2/p7-RLuc or mNG-p2/p7-NLuc), in the presence or absence of HIV-PR expression plasmids (40 ng). In parallel, cells were either mock transfected for background subtraction or transfected with BRET donor-only plasmid alone (pDESTnt-RLuc-UL44, 250 ng and pCDNA3-NLuc-UL44, 100 ng). At twenty-four hours p.t., culture medium was removed and cells gently washed with 1 mL of PBS. Cells were then resuspended in 290 µL of PBS, and 90 µL of each suspension was transferred in flat-bottom, tissue culture-treated 96-well microplates optimized for fluorescence (Costar, Arlington, VA, USA, #3916) or luminescence (Costar, #3917) spectral measurements. Spectra were acquired using a Varioskan^TM^ Lux multimode microplate reader (Thermo Fisher Scientific, Monza, Italy). YFP excitation spectra were acquired by exciting samples across the 420–542 nm interval with 1 nm steps, followed by emission acquisition at 560 nm. mNG excitation spectra were generated by exciting samples across the 420–529 nm interval with 1 nm steps, followed by emission acquisition at 545 nm. YFP emission spectra were acquired by excitation at 488 nm, followed by emission acquisition across the 506–616 nm interval with 1 nm steps. mNG emission spectra were acquired by excitation at 450 nm, followed by emission acquisition across the 468–618 nm interval with 1 nm steps. RLuc and NLuc emission spectra were acquired by repeatedly measuring light across the 400–575 nm interval with 5 nm steps for 45 min, using emission at 480 nm as a reference. Integrated measurements at specific wavelengths were then divided for the integrated measurements at 480 nm. Spectral curves were generated using GraphPad prism, using 5-point centered moving averages for relative intensities.

### 2.6. Bioluminescence Resonance Energy Transfer (BRET) Assays

BRET experiments were performed essentially as described in [18,19,20]. HEK293T cells were seeded and transfected as above. Transfections included 15 ng of plasmids encoding either HIV-1 PR BRET sensor probes (YFP-p2/p7-RLuc or mNG-p2/p7-NLuc) or their non-cleavable counterparts (YFP-RLuc or mNG-NLuc), in the presence or absence of HIV-PR expression plasmids (0–100 ng). In parallel, cells were either mock transfected for background subtraction or transfected with 5 ng of BRET donor-only plasmid alone (pDESTnt-RLuc-UL44 and pCDNA3-NLuc-UL44), to allow subtraction of BRET signals and calculation of BRET ratio relative to each BRET reporter probe. At twenty-four hours p.t., culture medium was removed and cells gently washed with 1 mL of PBS. Cells were then resuspended in 290 µL of PBS, and 90 µL of each suspension was transferred in triplicate to a black, flat-bottom, tissue culture-treated 96-well microplate (Costar, #3916). Signals were acquired using a VICTOR X2 Multilabel Plate Reader compatible with BRET measurements (Perkin Elmer, Waltham, MA, USA). Fluorescence signals relative to YFP (YFP Net) or mNG (mNG Net) emission were acquired using a fluorometric excitation filter (485 ± 14 nm band pass) and a fluorometric emission filter (535 ± 25 nm band pass). Luminometric readings were performed at 15′ after addition of h-Coelenterazine (h-CTZ; PJK) at a final concentration of 5 μM. Data were acquired for 1 sec/well, using a luminometric 535 ± 25 nm band pass emission filter (acceptor: YFP and mNG) and a luminometric 460 ± 25 nm emission filter (donor: RLuc and NLuc). Before reading, the plate was shaken for 1 sec at normal speed using a double orbit mode. After background subtraction using values relative to mock-transfected cells, BRET values were calculated as the ratio between the acceptor to donor signal for each, according to the formula: BRET value = (acceptor emission)/(donor emission). BRET ratios were calculated by subtracting the BRET value of the donor-only control from the BRET value of each corresponding BRET pair: BRET ratio = (BRET value)_BRET pair_—(BRET value)_donor only_. Alternatively, signals were acquired using a Varioskan^TM^ Lux multimode microplate reader (Thermo Fisher Scientific, Monza, Italy) for 1 sec/well, using a luminometric 530 ± 30 nm band pass emission filter (acceptor: YFP and mNG) and a luminometric 480 ± 20 nm emission filter (donor: RLuc and NLuc).

### 2.7. PR Inhibitors Screening Using mNG-p2/p7-NLuc

HEK293T cells were seeded and transfected as described above, with 15 ng of mNG-p2/p7-NLuc alone or in combination with the appropriate amount of pcDNA4/TO-PR. Six hours p.t., cell culture media was replaced with DMEM containing either DMSO or PR inhibitors at a final concentration of 10 μM and 100 μM. Twenty-four hours p.t., cells were processed for BRET measurements. To calculate the half-maximal inhibitory concentration (IC_50_) of active compounds, HEK293T were transfected as above and, 6 h p.t., treated with increasing concentrations of compounds (ranging from 0.001 to 100 μM). Cells were then processed for BRET assays as described above. IC_50_ values were calculated using GraphPad Prism 9 software (Graphpad Software Inc., La Jolla, CA, USA), by plotting each individual BRET ratio value to the logarithm of compound concentration. The data were interpolated using the “log(inhibitor) vs. response” function of GraphPad Prism 9 (Graphpad Software Inc.).

### 2.8. Statistical Analysis

Statistical analyses were performed using Graphpad Prism 9 (Graphpad Software Inc.). Data from BRET experiments were analyzed using one-way analysis of variance (ANOVA) and Tukey’s multiple-comparison post-test, or the unpaired Student’s *t*-test, with Welch’s correction. Differences between groups were considered to be significant at *p* < 0.05.

## 3. Results

### 3.1. Design of BRET-Based HIV-1 PR Reporters

**Figure 1:**
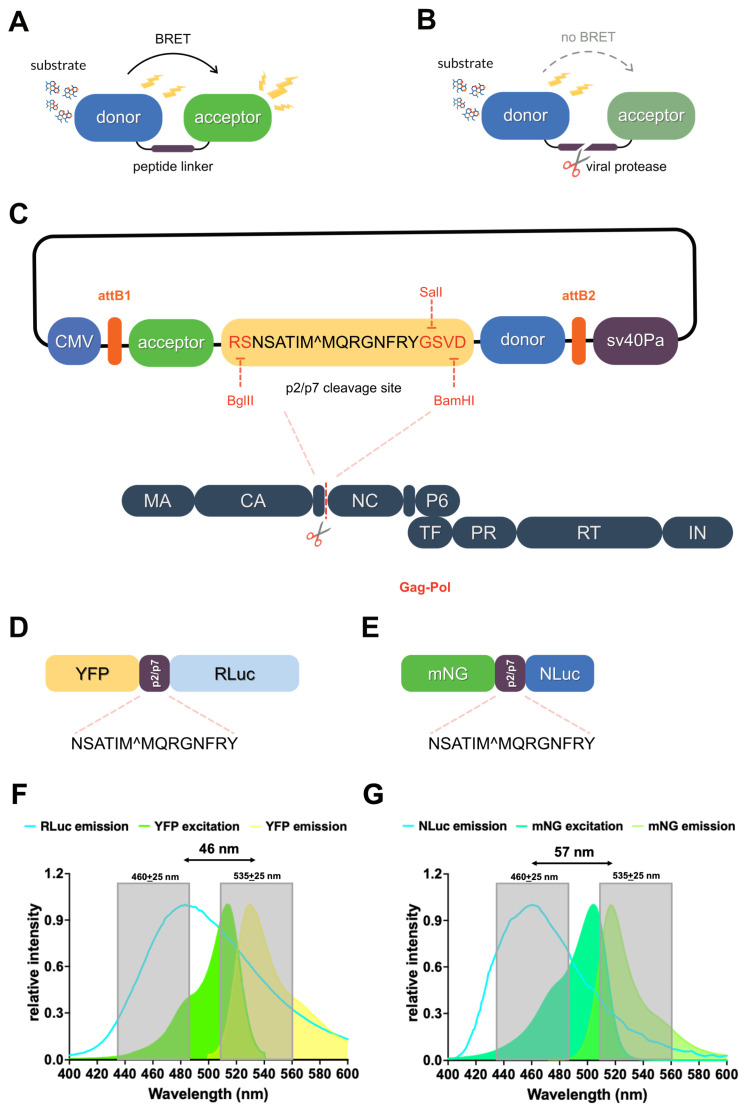
Design of flexible plasmids mediating the expression of HIV-1 PR BRET reporters. (**A**) Schematic representation of a BRET-based HIV-1 PR reporter. The reporter is a fusion protein comprising a bioluminescent donor and a fluorescent acceptor connected by a peptide linker containing an HIV-1 PR cleavage site. In the absence of HIV-1 PR, addition of the donor substrate results in efficient energy transfer and a high BRET signal, as donor and acceptor remain in close proximity (<10 nm). (**B**) Upon HIV-1 PR expression, cleavage of the linker separates donor and acceptor, leading to reduced energy transfer and a strong decrease in BRET signal. (**C**) Schematic representation of the plasmids encoding HIV-1 PR reporters generated in this study. CMV: human cytomegalovirus promoter, allowing strong constitutive expression in mammalian cells; attB1 and attB2: Gateway recombination sites, allowing the transfer of the comprised sequence to Gateway entry vectors containing attP sites; acceptor: fluorescent protein (YFP or mNG); p2/p7: cleavage site of HIV-1 gag-pol polyprotein; donor: bioluminescent protein (RLuc or NLuc); SV40pA: simian virus 40 poly adenylation signal. (**D**,**E**) Configuration of the YFP-p2/p7-RLuc (**D**), and the mNG-p2/p7-NLuc (**E**) reporters. (**F**,**G**) Donor emission (blue lines), as well as acceptor excitation and emission (colored curves) spectra, relative to the YFP-p2/p7-RLuc (**F**), and the mNG-p2/p7-NLuc (**G**) reporter are shown. The gray dashed boxes correspond to light passing through 460 ± 25 nm and 535 ± 25 nm emission filters, and the black double headed arrow indicates the distance between donor and acceptor emission peaks. Spectral properties of mNG, YFP, and NLuc were downloaded from FPbase (https://www.fpbase.org), whereas RLuc emission spectrum was downloaded from the dryad database (https://datadryad.org/dataset/doi:10.5061/dryad.rv15dv4gm, accessed on 23 July 2025). Curves were generated with Graphpad Prism.

We aimed to develop BRET-based reporters for monitoring viral proteases activity in living cells. In the absence of protease, the reporter would allow efficient energy transfer from its bioluminescent donor moiety to the fluorescent acceptor moiety, resulting in a high BRET ratio (Figure 1A). Protease activity would result in cleavage of the sensor, separating the donor and acceptor moiety, thereby disrupting energy transfer and yielding a low BRET ratio (Figure 1B). As a proof of concept, we generated plasmids encoding BRET-based HIV-1 PR reporters, in which the p2/p7 cleavage site was inserted between an acceptor (either YFP or mNG) and a donor (either RLuc or NLuc) moiety. The cleavage site is flanked by restriction sites, allowing for modular replacement with alternative protease recognition sequences via BglII/SalI digestion, or for generation of a non-cleavable control probe via BglII/BamHI digestion. Additionally, the reporter cassette is flanked by Gateway attB recombinant sites, enabling rapid exchange of promoter and expression system (Figure 1C). This approach allowed direct comparison between the standard HIV-1 PR BRET reporter based on YFP-RLuc (Figure 1D) and a novel mNG-NLuc version (Figure 1E), each endowed with specific spectral properties (Figure 1F,G).

### 3.2. Validation of BRET-Based HIV-1 PR Reporters in a Cellular Context

**Figure 2:**
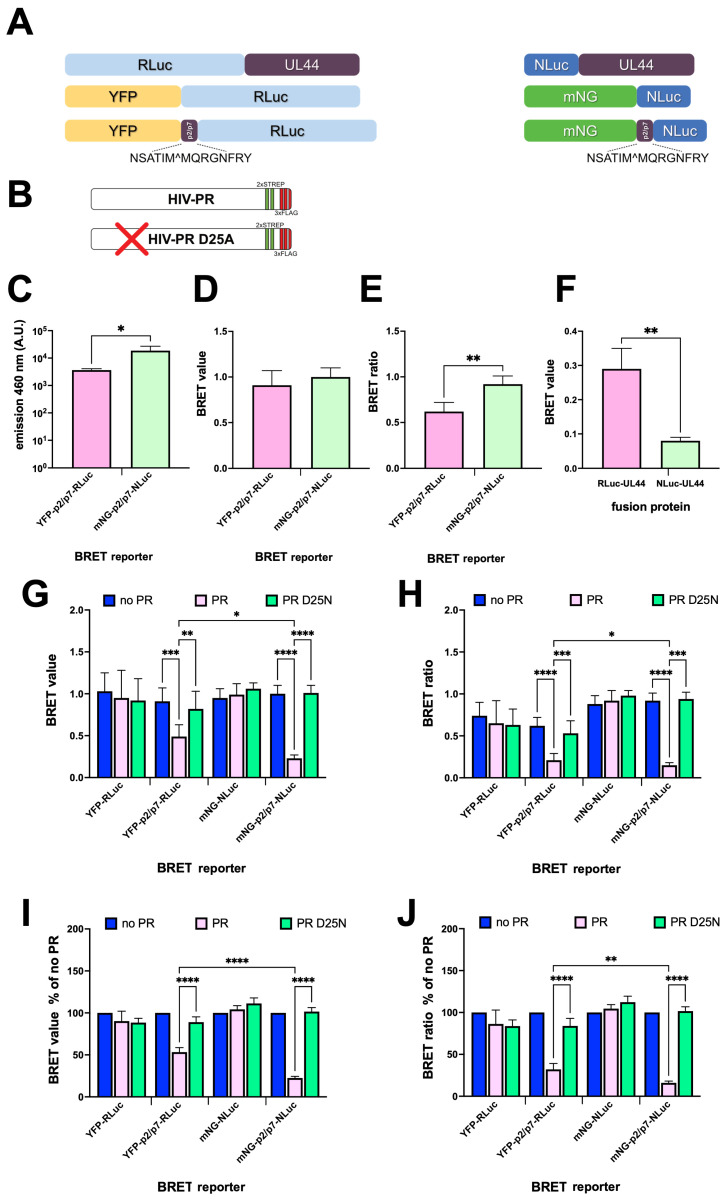
Development of HIV-1 PR BRET cellular reporters. HEK293T cells were seeded in a twenty-four-well plate and transfected to express the indicated fusion proteins alone (**A**) or in combination with either HIV-1 PR or its catalytically inactive derivative (**B**). Twenty-four hours p.t., cells were transferred to 96-well plates and subjected to BRET assays as described under the Materials and Methods section. The donor emission (**C**), the BRET value (**D**), and the BRET ratio (**E**) relative to YFP-p2/p7-RLuc and mNG-p2/p7-NLuc when expressed in the absence of HIV-1 PR are shown, along with the BRET values relative to RLuc-UL44 and NLuc-UL44 (**F**). (**G**–**J**) BRET values (**G**,**I**) and BRET ratios (**H**,**J**) calculated for the indicated reporter proteins expressed in the absence of HIV-1 PR (no PR, blue columns) or in the presence of either wild-type HIV-1 PR (PR, pink columns) or its catalytically inactive derivative bearing the D25N substitution (D25N, sea-green columns) are shown as raw values (**G**,**H**) or as percentages relative to the corresponding values measured in the absence of HIV-1 PR (**I**,**J**). All data shown are mean ± standard deviation of the mean relative bioluminescent signals acquired 15 min after addition of h-CTZ, from three independent experiments performed in triplicate, along with results of the Welch *t*-test for statistical significance. *: *p* < 0.05; **: *p* < 0.01; ***: *p* < 0.001; ****: *p* < 0.0001.

In order to compare the two different BRET settings, we transfected HEK293T cells with plasmids expressing each BRET reporter (Figure 2A), in the presence or absence of plasmids encoding either HIV-1 PR or a catalytically impaired derivative carrying the D25N substitution within the catalytic site (Figure 2B). Cells were also transfected with plasmids expressing the BRET donor alone—to allow calculation of the BRET ratio (see Materials and Methods section)—and the control BRET reporters lacking the p2/p7 cleavage site (Figure 2A). Twenty-four hours p.t., cells were transferred to a 96-well plate and fluorescence emission was quantified upon excitation at 488 nm (Supplementary Figure S1A). Subsequently, h-CTZ was added to each well and luminometric measurements were performed using the 460 ± 25 nm (Supplementary Figure S1B) and 535 ± 25 nm (Supplementary Figure S1C) emission filters, which allowed calculation of BRET values and BRET ratios. Importantly, addition of h-CTZ resulted in approximately 100-fold higher emission for NLuc compared to RLuc at both 460 and 535 nm (Figure 2C), indicating that the mNG-NLuc-based BRET reporter allows generation of detectable luminescent signals at lower expression levels compared to YFP-RLuc upon addition of the same substrate. In the absence of PR expression, BRET values and BRET ratios for mNG-p2/p7-NLuc were higher than those for YFP-p2/p7-RLuc (1.00 vs. 0.92, and 0.91 vs. 0.62, respectively; see Figure 2D,E); this demonstrated that both BRET reporters are functional in terms of energy transfer and that mNG-NLuc is more efficient in this respect than YFP-RLuc. Furthermore, the BRET value relative to NLuc-UL44 was significantly lower than that of RLuc-UL44 (0.08 vs. 0.27), suggesting lower bleed-through due to donor emission in the acceptor emission range for mNG-NLuc compared to YFP-RLuc (Figure 2F). Importantly, expression of HIV-1 PR significantly reduced both the BRET value (Figure 2G) and the BRET ratio (Figure 2H) of the reporters containing the p2/p7 cleavage site, but not of those lacking it. Additionally, no decrease was detected upon co-expression of the catalytically inactive HIV-1 PR D25N, further supporting the specificity of probe cleavage (Figure 2G, cyan columns). Expression of HIV-1 PR caused a stronger reduction in both BRET values (Figure 2G) and ratios (Figure 2H) for mNG-p2/p7-NLuc to YFP-p2/p7-RLuc. A stronger reduction was also observed for normalized BRET measurements (Figure 2I,J).

### 3.3. Spectral Properties of the YFP-p2/p7-RLuc and mNG-p2/p7-NLuc BRET Reporters

**Figure 3:**
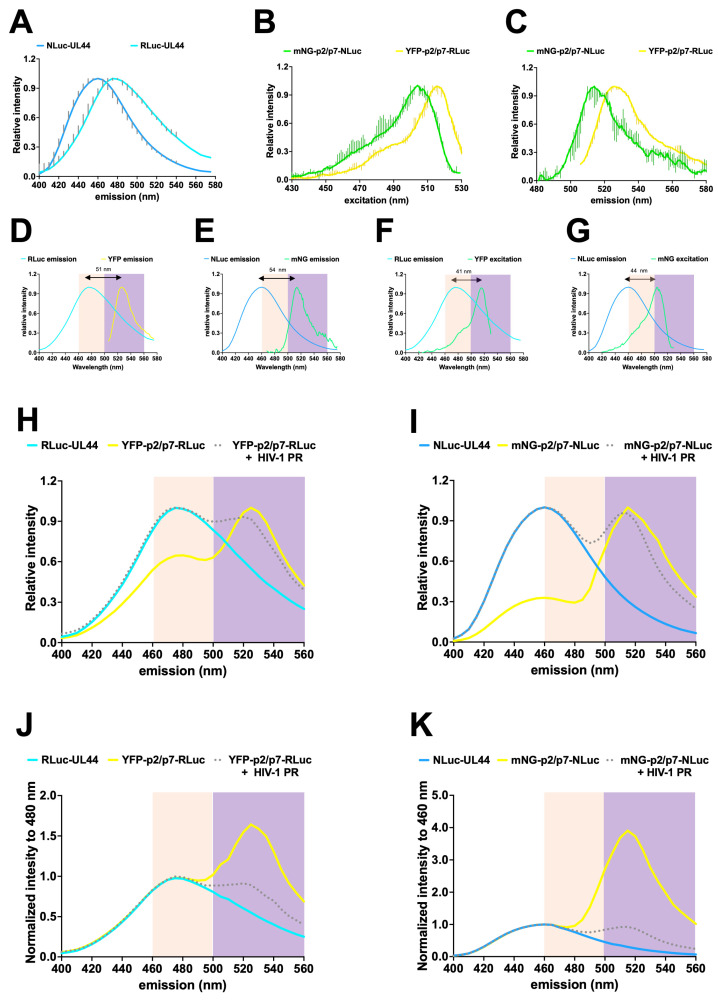
Full spectral properties of HIV-1 BRET reporters developed in this study. HEK293T cells were transfected to express the BRET reporters YFP-p2/p7-RLuc and mNG-p2/p7-NLuc in the absence or presence of HIV-1 PR, or the bioluminescent controls RLuc-UL44 or NLuc-UL44. Twenty-four hours post-transfection, cells were processed for fluorescent and luminometric spectral acquisition (**A**–**K**), using a Varioskan LUX plate luminometer as described in the Materials and Methods section. (**A**) RLuc (RLuc-UL44) and NLuc (NLuc-UL44) emission spectra. Data shown are median values ± 95% CI relative to three independent experiments performed in duplicate. (**B**) YFP-p2/p7-RLuc and mNG-p2/p7-NLuc excitation spectra. Data shown are median values ± 95% CI relative to three independent experiments. (**C**) YFP-p2/p7-RLuc and mNG-p2/p7-NLuc emission spectra. Data shown are median values ± 95% CI relative to three independent experiments. (**D**) Spectral overlap between RLuc and YFP emission spectra. (**E**) Spectral overlap between NLuc and mNG emission spectra. (**F**) Spectral overlap between RLuc emission and YFP excitation spectra. (**G**) Spectral overlap between NLuc emission and mNG excitation spectra. (**H**) Emission spectra relative to YFP-p2/p7-RLuc expressed in the absence or in the presence of HIV-1 PR, superimposed on the emission spectrum of RLuc-UL44 for comparison. (**I**) Emission spectra relative to mNG-p2/p7-NLuc expressed in the absence or in the presence of HIV-1 PR, superimposed on the emission spectrum of NLuc-UL44 for comparison. (**J**) Emission spectra relative to YFP-p2/p7-RLuc expressed in the absence or in the presence of HIV-1 PR, superimposed on the emission spectrum of RLuc-UL44 for comparison, with emission at 480 nm set to 1. (**K**) Emission spectra relative to mNG-p2/p7-NLuc expressed in the absence or in the presence of HIV-1 PR, superimposed on the emission spectrum of NLuc-UL44 for comparison, with emission at 460 nm set to 1. In panels (**D**–**K**), the emission windows corresponding to the 480 ± 20 nm and 530 ± 30 nm filters are highlighted in pink and violet, respectively.

The enhanced performance of the mNG-p2/p7-NLuc reporter compared to YFP-p2/p7-RLuc is likely due to the better spectral properties of the NLuc/mNG BRET pair relative to YFP/RLuc (Figure 1F,G and Figure 2D). Although the differences in emission spectra between NLuc with its specific substrate, furimazine, and RLuc with coelenterazine (CTZ) have been extensively characterized [13], little is known about their emission properties when h-CTZ is used as a common substrate. To better assess the differences in energy transfer and emission properties of the YFP-RLuc and mNG-NLuc BRET pairs in the presence of h-CTZ, fluorescence and bioluminescent emission spectra of both reporters were measured in living HEK293T cells expressing the corresponding fusion proteins (Figure 3). Notably, the bioluminescent emission spectrum of NLuc-UL44 (peak at 460 nm) was shifted towards lower wavelengths compared to RLuc-UL44 (peak at 475 nm), resulting in a significantly reduced relative emission at 530 nm (Figure 3A). Furthermore, both the fluorescent excitation (Figure 3B) and emission (Figure 3C) spectra of mNG-p2/p7-NLuc were similarly shifted towards lower wavelengths compared to YFP-p2/p7-RLuc. Overall, we measured a slightly greater distance between NLuc emission peak and mNG emission (Figure 3E) and excitation peaks (Figure 3G) compared to RLuc and YFP (Figure 3D,F), thus confirming the possibility of using h-CTZ as a common substrate for comparing YFP-RLuc and mNG-NLuc BRET reporters. Superimposition of reporter emission spectra in the absence or presence of HIV-1 PR (Figure 3H–K and Figure S2A,B) revealed that in the absence of HIV-1 PR, energy transfer was significantly more efficient for mNG-p2/p7-NLuc (Figure 3I,K and Figure S2B, yellow curves) than for YFP-p2/p7-RLuc (Figure 3H,J and Figure S2A, yellow curves). Remarkably, in both cases, the expression of HIV-1 PR reduced, but energy transfer was not completely abolished (Figure 3 and Figure S2, compare blue/cyan and gray dotted curves), indicating that neither reporter was completely cleaved. The same samples were used to perform BRET measurements using 480 ± 20 and 530 ± 30 nm luminometric filters slightly different from those used originally (460 ± 25 and 535 ± 25 nm), and the difference in performance between the mNG-p2/p7-NLuc and the YFP-p2/p7-RLuc reporter was even greater than that measured previously (Supplementary Figure S2C–F).

### 3.4. The BRET Reporters Are Cleaved by HIV-1 PR

**Figure 4:**
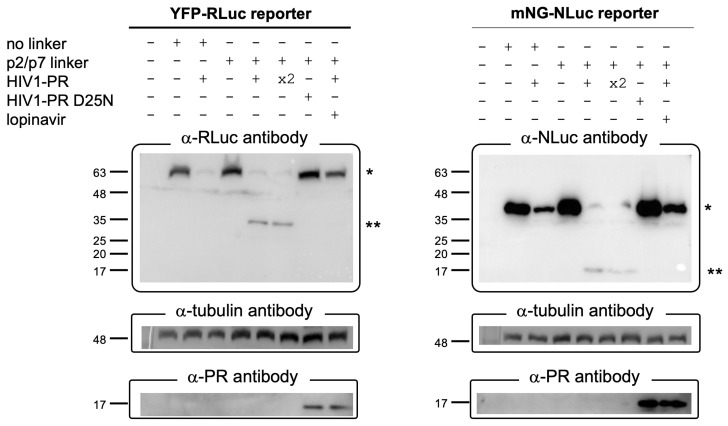
Validation of cleavage of BRET reporters by HIV-1 PR. HEK293T cells were seeded in 6-well plates. The day after, cells were transfected to express the indicated reporters either in the absence (−) or in the presence (+) of equal amounts of plasmids mediating the expression of HIV-1 PR or its catalytically inactive derivative D25N. Alternatively, cells were transfected with a double amount (×2) of the HIV-1 PR expressing plasmid. Six hours later, transfection reactions were removed and replaced with either complete medium or complete medium containing LOP (10 µM). Twenty-four hours p.t., cells were lysed and cell lysates used for SDS-PAGE Western blotting analysis followed by incubation of the indicated antibodies. * = uncleaved reporter; ** = cleaved reporter.

To verify whether the decrease in BRET observed for the HIV-PR reporter was due to cleavage of the p2/p7 linker by the catalytically active HIV-1 PR, we performed Western blotting experiments. To this end, HEK293T cells were transfected with plasmids encoding either the YFP-p2/p7-RLuc or mNG-p2/p7-NLuc reporter, or the control constructs lacking the p2/p7 linker, in the presence or absence of plasmids expressing the HIV-1 PR or its catalytically inactive mutant D25N. As an additional control, cells expressing the reporters and the HIV-1 PR were also treated with LOP (10 µM). As expected, in the absence of HIV-1 PR both YFP-RLuc and YFP-p2/p7-RLuc could be detected by the a-RLuc antibody as a single band with an apparent molecular weight of ~63 kDa (Figure 4). Importantly, a slower migrating band of approximately 36 kDa could be detected when YFP-p2/p7-RLuc was co-expressed with HIV-1 PR, corresponding to the RLuc fragment released after cleavage within the p2/p7 linker. No cleaved RLuc could be detected after expression of YFP-RLuc in the presence of HIV-1 PR, or when YFP-p2/p7-RLuc was expressed in the presence of either HIV-1 PR and LOP or the catalytically impaired HIV-1 PR D25N derivative (Figure 4, left panel). Very similar results were obtained after detection of NLuc from cell lysates transfected with mNG-NLuc and mNG-p2/p/-NLuc, with the band corresponding to uncleaved and cleaved reporters being detected at apparent molecular weight of 45 kDa and 17 kDa, respectively (Figure 4, right panel). Intriguingly, HIV-1 PR expression significantly decreased expression of the reporters, regardless of the presence of the p2/p7 cleavage site, most likely as a consequence of its ability to interfere with cap-dependent translation [2,31]. Accordingly, HIV-1 PR could not be detected in cell lysates, even when an excess of plasmid was used for transfection, unless its enzymatic activity was inhibited by LOP, or by introduction of the D25N substitution (Figure 4, bottom panels). Moreover, a fraction of each reporter remained uncleaved upon transfection with HIV-1 PR, consistent with BRET measurements reported in Figure 2G–J and Figure 3H,I and Supplementary Figure S3.

### 3.5. mNG-p2/p7-NLuc Detects HIV-1 PR Activity in a Dose Dependent Manner

**Figure 5:**
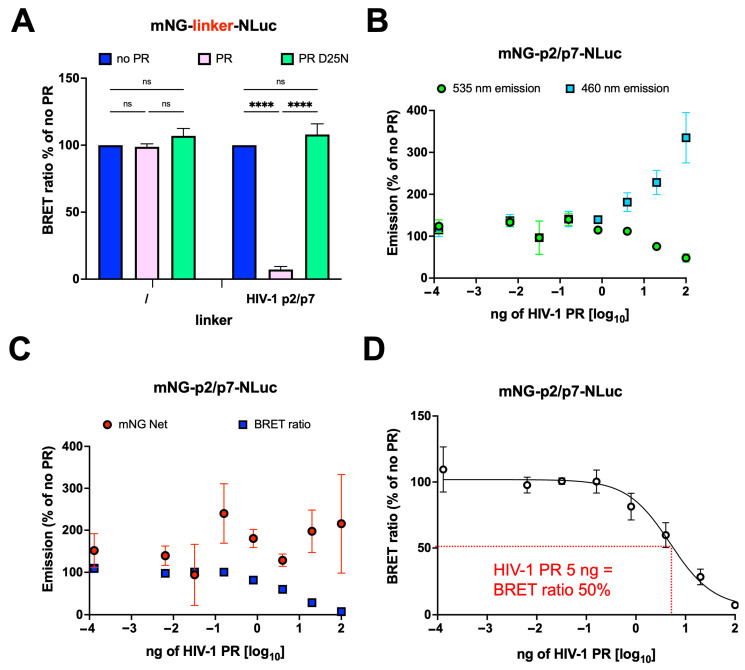
HIV-1 PR dose-dependent cleavage of the mNG-p2/p7-NLuc reporter in living cells. HEK293T cells were transfected with a fixed amount of mNG-p2/p7-NLuc expression plasmid in the absence or presence of increasing amounts of HIV-1 PR expression plasmid. Twenty-four hours p.t., cells were processed for BRET measurements as described in the Materials and Methods section. (**A**) BRET ratios from cells transfected to express either mNG-NLuc (left) or mNG-p2/p7-NLuc (right), individually (blue columns), and in the presence of maximum amounts of plasmids encoding either the wild-type (pink columns) or the catalytically inactive D25N (cyan columns) HIV-1 PR are shown. All data are expressed as mean percentages relative to measurements obtained in the absence of PR, along with standard deviation of the mean from three experiments performed in triplicate, and results of two-way ANOVA test for significance. **** = *p* < 0.0001; ns = not significant. (**B**,**C**) Luminescent emission measured using 535 and 460 nm emission filters (**B**), mNG Net and BRET ratios (**C**) from cells transfected to express mNG-p2/p7-NLuc in the presence of increasing amounts of HIV-1 PR-expressing plasmid are shown. (**D**) Nonlinear regression was used to calculate the amount of HIV-1 PR-expressing vector required to reduce the BRET ratio by 50%. All data are expressed as mean percentages relative to measurements obtained in the absence of PR, along with standard deviation of the mean relative to three experiments performed in triplicate.

Based on our results, we concluded that the mNG-p2/p7-NLuc BRET HIV-1 PR reporter is superior to the classical YFP-p2/p7-RLuc reporter. Therefore, only mNG-p2/p7-NLuc was used for subsequent experiments. We next investigated whether the reduction in BRET ratio observed with mNG-p2/p7-NLuc upon expression of HIV-1 PR is dose-dependent. To this end, HEK293T cells were transiently transfected with a fixed amount of mNG-p2/p7-NLuc (15 ng), in the presence of increasing amounts of HIV-1 PR expression plasmid (0–100 ng). Fixed amounts of mNG-NLuc (15 ng) and HIV-1 PR D25N (100 ng) were also expressed as negative controls. Importantly, transfection with the highest amount of HIV-1 PR expressing plasmid almost completely reduced the BRET ratio relative to mNG-p2/p7-NLuc but did not affect the BRET ratio of mNG-NLuc (Figure 5A). Moreover, expression of HIV-1 PR D25N did not affect the BRET ratio of mNG-p2/p7-NLuc, further confirming reporter specificity (Figure 5A). Notably, increasing amounts of HIV-1 PR led to a dose-dependent decrease in mNG-p2/p7-NLuc emission at 535 nm, which was mirrored by an increase in emission at 460 nm (Figure 5B and Figure S3), suggesting dose-dependent cleavage of mNG-p2/p7-NLuc. Accordingly, BRET ratio similarly decreased in an HIV-1 PR dose-dependent fashion, reaching >90% reduction in the presence of 100 ng of HIV-1 PR expression plasmid (Figure 5C and Figure S3). In the tested conditions, only a minimal decrease in mNG-mediated fluorescence was observed, indicating a lack of cytotoxicity (Figure 5C and Figure S3). Finally, by transforming HIV-1 PR plasmid amount to logarithmic scale and applying nonlinear regression to the BRET ratios, we could calculate the HIV-1 PR plasmid amount required to mediate a 50% decrease in BRET ratio (5 ng, Figure 5D).

### 3.6. mNG-p2/p7-NLuc as a Tool for Antiviral Drug Discovery

**Figure 6:**
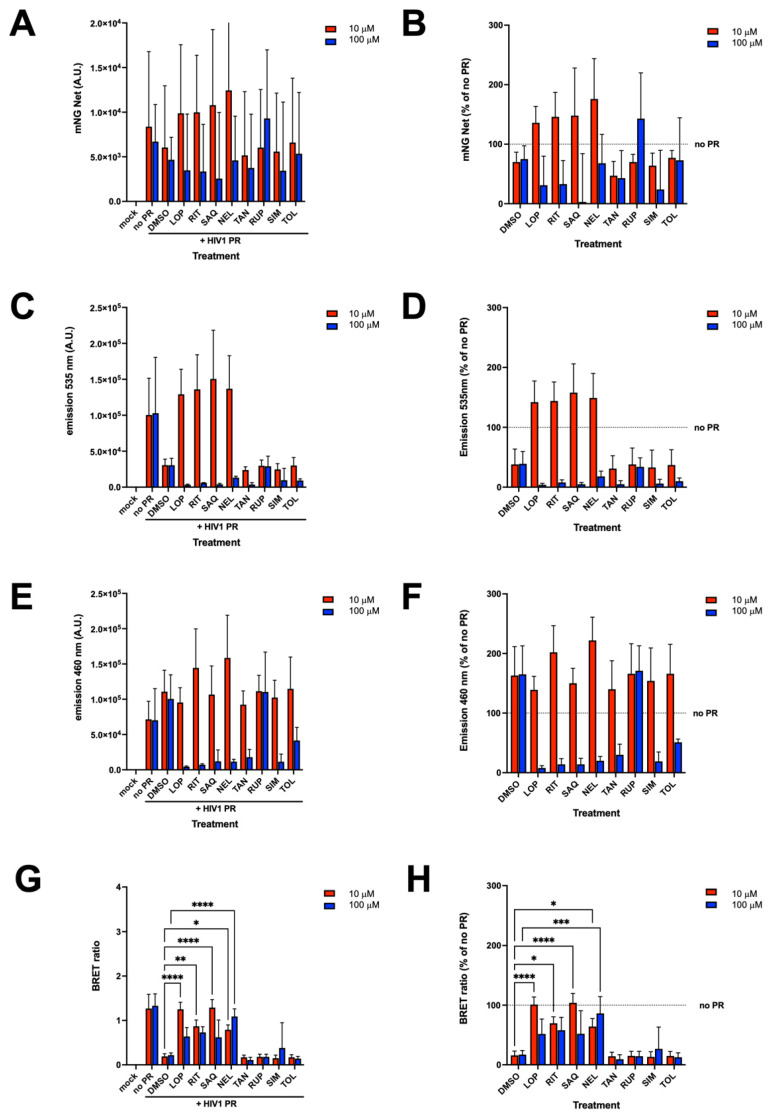
mNG-p2/p7-NLuc allows screening of HIV-1 PR inhibitors. HEK293T cells were transfected to express the mNG-p2/p7-NLuc reporter in the absence or presence of HIV-1 PR. Six hours p.t., media was replaced with fresh media containing either DMSO or the indicated inhibitors at two different concentrations (10 µM or 100 µM). The mNG fluorescence emission (**A**,**B**), as well as the luminescence emission measured using a 535 nm (**C**,**D**) and a 460 nm (**E**,**F**) emission filter are shown. Data were used to calculate the BRET ratio (**G**,**H**) relative to the indicated conditions. Data shown are mean ± standard deviation of the mean relative to four independent experiments performed in triplicate, with horizontal dashed lines indicating values obtained in the absence of HIV-1 protease (no PR), along with results of two-way ANOVA test for significance. * = *p* < 0.05; ** = *p* < 0.01; *** = *p* < 0.001; **** = *p* < 0.0001.

We next investigated whether the BRET reporter we developed in this study could be applied to screen compounds able to inhibit HIV-1 PR activity. To this end, HEK293T cells were transfected with fixed amounts of plasmid expressing mNG-p2/p7-NLuc (15 ng) in the presence or absence of plasmid encoding for HIV-1 PR corresponding to four times the amount required to cleave 50% of the reporter (20 ng). Six hours p.t., media was replaced with DMEM containing either DMSO only or two different concentrations of a panel of protease inhibitors (Figure 6). These include the well-known HIV-1 PR inhibitors RIT, LOP, SAQ and NEF, as well as the Hepatitis C virus NS3/4A PR inhibitor SIM, the rhinovirus 3C PR inhibitor RUP, and the flaviviral PR inhibitors TAN and TOL. Each compound was tested at 10 and 100 µM. The effect of inhibitors on cell viability was investigated using mNG fluorescence (mNG Net) as a surrogate and revealed that, with the exception of RUP and TOL, all tested inhibitors reduced cell viability at 100 µM (Figure 6A,B, compare red and blue columns). As expected, in the absence of PR inhibitors, co-expression of mNG-p2/p7-NLuc along with HIV-1 PR resulted in a decrease in bioluminescent emission at 535 nm (Figure 6C,D) and an increase at 460 nm (Figure 6E,F) after addition of h-CTZ. This is in accordance with the observed reduction in BRET ratio due to mNG-p2/p7-NLuc cleavage by HIV-1 PR (Figure 6G,H). Interestingly, incubation with HIV-1 PR-specific inhibitors promptly restored BRET ratios to levels comparable to those calculated in the absence of HIV-1 PR, whereas incubation with other inhibitors did not (Figure 6G,H).

### 3.7. The mNG-p2/p7-NLuc Reporter Allows Measuring the Potency of HIV-1 PR Inhibitors in Living Cells

**Figure 7:**
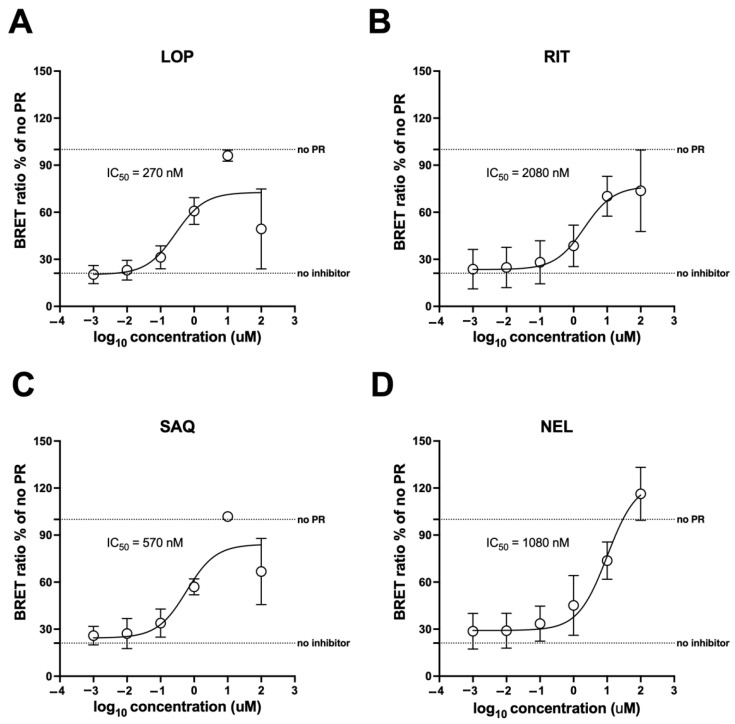
Calculation of IC_50_ values relative to HIV-1 PR inhibitors in living cells using mNG-p2/p7-NLuc. HEK293T cells were transfected to express the mNG-p2/p7-NLuc reporter in the absence or presence of HIV-1 PR. Six hours p.t., media was replaced with fresh media containing either DMSO or increasing concentrations of lopinavir (LOP; (**A**)), ritonavir (RIT; (**B**)), saquinavir (SAQ; (**C**)) and nelfinavir mesylate (NEL; (**D**)). Twenty-four hours p.t., cells were transferred to 96-well plates for BRET assays as described in the Materials and Methods section. BRET ratios, normalized to those obtained in the absence of PR, were used for data fitting using GraphPad PRISM, as described in the Materials and Methods section, to calculate IC_50_ values. Data shown are mean ± standard deviation of the mean relative to three independent experiments performed in triplicate, with horizontal dashed lines indicating values obtained in the absence of HIV-1 protease (no PR) or in the presence of HIV-1 protease but in the absence of inhibitors (no inhibitor).

We investigated whether the mNG-p2/p7-NLuc reporter could serve as a tool for quantifying IC_50_ values of HIV-1 protease inhibitors in living cells. To this end, HEK293T cells were transfected with a plasmid expressing mNG-p2/p7-NLuc in the presence or absence of plasmid encoding for HIV-1 PR. Six hours p.t., media was replaced with DMEM containing either DMSO only or increasing concentrations of HIV-1 PR inhibitors RIT, LOP, SAQ, and NEF (range 0.001–100 µM). The effect of inhibitors on cell viability was investigated using mNG fluorescence (mNG Net) as a surrogate and confirmed that all compounds affected cell viability at 100 µM (Supplementary Figure S4). A dose-dependent restoration of the BRET ratio was observed, indicating progressive inhibition of HIV-1 PR activity by the tested compounds (Supplementary Figure S4). Data fitting allowed estimation of the IC_50_ for each inhibitor in living HEK293T cells (Figure 7). Under such settings, LOP was the most potent PR inhibitor (Figure 7A; IC_50_ = 270 nM), followed by SAQ (Figure 7C; IC_50_ = 570 nM), NEL (Figure 7D; IC_50_ = 1080 nM), and RIT (Figure 7B; IC_50_ = 1800 nM). Therefore, these results show that the mNG-p2/p7-NLuc reporter is a reliable tool for quantifying the potency of HIV-1 PR inhibitors in living cells.

## 4. Discussion

Viral infections cause significant morbidity and mortality worldwide. Despite the tremendous impact on human health of highly active and well-tolerated antivirals [3], no specific antiviral drugs have been approved for the treatment of the majority of viral infections [32,33], including emerging respiratory pathogens such as enteroviruses [34], and vector-borne pathogens such as flaviviruses [35], which pose a serious threat to global health. Unfortunately, current assays for viral PR inhibitors discovery are mostly performed in vitro and are thus endowed with several drawbacks, including the inability to take into account cell permeability and toxicity and to identify compounds requiring cellular activation. To overcome such limitations, several cell-based assays are beginning to be developed, based on the simultaneous expression of the viral PR of interest, a reporter capable of generating a specific signal upon cleavage, and of potential viral PR inhibitors. Each of these assays has specific advantages and disadvantages, including low signal-to-noise ratios, low sensitivity to viral protease inhibitors, and complex data analysis [8,9,10]. Among them, BRET is rapidly emerging as a valuable tool in biomedical research, due to its high sensitivity, reproducibility, and independence from complex data analysis, features which make it easily suitable for large high-throughput screening projects [17,36,37,38,39]. Furthermore, recent development of NLuc, a novel, highly versatile luciferase further expanded BRET flexibility and reliability [14]. We decided to compare the traditional YFP-RLuc BRET pair to mNG-NLuc for the detection of viral PR activity in living cells, using the extremely well-characterized HIV-1 PR as a starting point. To this end, we developed Mammalian expression vectors encoding two types of BRET reporters, in which a fluorescent protein (YFP or mNG) is linked to a bioluminescent donor (RLuc and NLuc, respectively) via the HIV-1 PR cleavage site p2/p7. These plasmids were designed to be compatible with the Gateway cloning technology for easy transfer to other expression systems, and additionally, they were designed to allow the rapid adaptation to different viral proteases by replacing the cleavage site region using BamHI and SalI restriction enzymes (Figure 1). The performance of the two sensors was compared after adding the substrate h-CTZ, which can be oxidized by both luciferases (14). Under these conditions, a tenfold higher emission intensity using a 460 + 25 nm emission filter was detected for NLuc compared to RLuc (Figure 2C). Furthermore, the narrower emission spectrum of NLuc compared to that of RLuc after the addition of h-CTZ (Figure 3A), and its greatly reduced emission in the wavelength range of filters commonly used to acquire BRET acceptor emission (Figure 1F,G and Figure 3D,E) allowed better spectral separation between the emission peaks of donor and acceptor. Accordingly, the background BRET signal measured for NLuc-UL44 was significantly lower than that measured for RLuc-UL44, when using standard pairs of emission filters such as 460 ± 25 and 535 ± 25 nm (Figure 2F) as well as 480 ± 20 and 530 ± 30 nm (Supplementary Figure S2E). As a consequence, BRET ratios were significantly higher for the mNG-p2/p7-NLuc reporter compared to YFP-p2/p7-RLuc (Figure 2H and Figure S2F). Furthermore, the higher quantum yield of mNG compared to YFP resulted in noticeably stronger emission of mNG-p2/p7-NLuc in the 530 nm range compared to YFP-p2/p7-RLuc (Figure 3J,H and Figure S2A,B).

Expression of HIV-1 PR, but not its catalytically inactive derivative bearing the D25N substitution, caused a significant decrease in BRET values and ratios for both YFP-p2/p7-RLuc and mNG-p2/p7-NLuc, but not for the control YFP-RLuc and mNG-NLuc reporters (Figure 2E–J), due to specific cleavage of the p2/p7 linker (Figure 4). Although both YFP-p2/p7-RLuc and mNG-p2/p7-NLuc reporters were efficiently cleaved by HIV-1 PR, the BRET ratio of mNG-p2/p7-NLuc decreased to a greater extent due to the superior spectral separation between mNG and NLuc compared to YFP and RLuc. In light of these findings, we conclude that the mNG-p2/p7-NLuc reporter is superior to YFP-p2/p7-RLuc. Despite expression of HIV-1 PR strongly reducing BRET ratios, those did not reach background levels (Figure 2H,J and Figure 5A and Supplementary Figure S2F), indicating that neither mNG-p2/p7-NLuc nor YFP-p2/p7-RLuc was completely cleaved by the viral protease, as confirmed by detection of a small fraction of uncleaved BRET reporter by Western blotting (Figure 4). The C-terminal 2 × StrepTagII–TEV–3 × FLAG, owing to its low isoelectric point, likely contributes to the reduced HIV-1 PR activity observed here [24]. Future work will evaluate tag removal or alternatives that restore native termini.

Recent studies described a similar HIV-1 PR reporter, consisting of RLuc and hGFP2 linked together via the HIV-1 p2/p7 sequence. Its utility has been validated using inhibitors such as Amprenavir and Saquinavir, confirming that BRET reporters can be used as tools for the identification of specific inhibitors [12]. Similarly, the validity of our mNG-p2/p7-NLuc reporter was confirmed through the screening of eight PR inhibitors either specific for HIV-1 PR (LOP, RIT, NEL and SAQ) or other viral PRs (TOL, TAN, RUP and SIM). As expected, treatment with HIV-1 PR inhibitors, but not with other inhibitors, increased the BRET ratio of mNG-p2/p7-NLuc in the presence of HIV-1 PR (Figure 6G,H). These results are in line with what was observed for a FRET-based biosensor in the presence of HIV-1 PR [40]. The dose-dependent curves relating to the four HIV-1 PR inhibitors LOP, SAQ, NEL, and RIT allowed us to obtain IC_50_ values of 270 nM, 570 nM, 1080 nM, and 1800 nM, respectively. These values are higher than the EC_50_ values reported for inhibition of HIV-1 replication in cell culture [41]. This may be due to variations in experimental conditions, such as the cell line used or the duration of drug treatment. Discrepancies between EC_50_ values were also observed in other studies where the antiviral activity of SAQ was evaluated in lymphoblastoid and monocytic cell lines and in peripheral blood lymphocytes in cell cultures, with EC_50_ ranging from 1 to 30 nM. Indeed, the antiviral effect of a PR inhibitor might also depend on the ability to inhibit other viral targets, or to interfere with cellular functions required for other steps of the virus life cycle [42]. Finally, it must be mentioned that partial HIV-1 PR inhibition might be sufficient to cause a complete block in viral replication. Similarly, IC_50_ values higher than those measured in vitro were reported for specific HIV-1 PR inhibitors in a T-cell line engineered to allow GFP expression upon HIV-1 PR inhibition [42]. In that study, the IC_50_ against HIV-1 of Atazanavir, Lopinavir, Indinavir or Tipranavir was estimated to be 1-to-10 nM, 10-to-50 nM, 100-to-500 nM, and 500-to-1000 nM, respectively. It is important to note that all BRET reporters were transiently expressed under the CMV immediate early promoter (Figure 1C), whose strong constitutive activity in mammalian cells can alter apparent protease–inhibitor dynamics. This limitation could be alleviated by employing stable cell lines expressing the reporters from weaker promoters, for instance, the Herpes simplex virus thymidine kinase promoter [43]. In summary, the mNG-p2/p7-NLuc reporter is an effective and robust tool for detecting HIV-1 PR activity in cells and for identifying viral protease inhibitors. The reporter’s design gives it high sensitivity, which represents an important advantage compared to other sensors reported in the literature. Furthermore, the mNG-p2/p7-NLuc reporter allowed the detection of HIV-1 PR activity at expression levels that were non-toxic to the cells. A great advantage of our experimental design is the possibility to easily switch the specificity of the reporter by replacing the p2/p7 cleavage site with peptide linkers targeted by proteases encoded by different viruses, and future work in our laboratory is heading in this direction.

## Data Availability

All data are available from Research Data Unipd (https://researchdata.cab.unipd.it/id/eprint/1661, accessed on 18 October 2025).

## Supplementary Materials

The following supporting information can be downloaded at https://www.mdpi.com/article/10.3390/v17101391/s1, Figure S1: Development of HIV-1 PR BRET cellular biosensors; Figure S2: Non-normalized emission spectra and BRET measurements corresponding to Figure 3; Figure S3: HIV-1 PR dose-dependent cleavage of the mNG-p2/p7-NLuc biosensor in living cells; Figure S4: Assessment of HIV-1 PR inhibitors using mNG-p2/p7-NLuc.

## Abbreviations

The following abbreviations are used in this manuscript:

BRET	Bioluminescent resonance energy transfer
RLuc	Renilla luciferase
NLuc	Nanoluc luciferase
h-CTZ	h-coelenterazine
mNG	mNeonGreen
HIV-1	Human Immunodeficiency Virus 1
DMEM	Dulbecco’s modified Eagle’s medium
FBS	Fetal bovine serum
IC_50_	Half-maximal inhibitory concentration

## References

[1] Borges P.H.O., Ferreira S.B., Silva F.P. (2024). Recent Advances on Targeting Proteases for Antiviral Development. Viruses.

[2] Centazzo M., Manganaro L., Alvisi G. (2023). Cellular Targets of HIV-1 Protease: Just the Tip of the Iceberg?. Viruses.

[3] De Clercq E. (2007). The design of drugs for HIV and HCV. Nat. Rev. Drug Discov..

[4] Manns M.P., von Hahn T. (2013). Novel therapies for hepatitis C—One pill fits all?. Nat. Rev. Drug Discov..

[5] Ohsaki Y., Sasaki T., Umekage Y., Yanada H., Ishikawa M., Yoshida R. (2025). Real-World Treatment Outcomes in the First and Subsequent Coronavirus Disease 2019 (COVID-19) Hospital Clusters. Cureus.

[6] Hong S.J., Resnick S.J., Iketani S., Cha J.W., Albert B.A., Fazekas C.T., Chang C.W., Liu H., Dagan S., Abagyan M.R. (2025). A multiplex method for rapidly identifying viral protease inhibitors. Mol. Syst. Biol..

[7] Ihssen J., Faccio G., Yao C., Sirec T., Spitz U. (2021). Fluorogenic in vitro activity assay for the main protease M(pro) from SARS-CoV-2 and its adaptation to the identification of inhibitors. STAR Protoc..

[8] Arias-Arias J.L., MacPherson D.J., Hill M.E., Hardy J.A., Mora-Rodriguez R. (2020). A fluorescence-activatable reporter of flavivirus NS2B-NS3 protease activity enables live imaging of infection in single cells and viral plaques. J. Biol. Chem..

[9] Pahmeier F., Neufeldt C.J., Cerikan B., Prasad V., Pape C., Laketa V., Ruggieri A., Bartenschlager R., Cortese M. (2021). A Versatile Reporter System To Monitor Virus-Infected Cells and Its Application to Dengue Virus and SARS-CoV-2. J. Virol..

[10] van der Linden L., Ulferts R., Nabuurs S.B., Kusov Y., Liu H., George S., Lacroix C., Goris N., Lefebvre D., Lanke K.H. (2014). Application of a cell-based protease assay for testing inhibitors of picornavirus 3C proteases. Antivir. Res..

[11] Bacart J., Corbel C., Jockers R., Bach S., Couturier C. (2008). The BRET technology and its application to screening assays. Biotechnol. J..

[12] Hu K., Clement J.F., Abrahamyan L., Strebel K., Bouvier M., Kleiman L., Mouland A.J. (2005). A human immunodeficiency virus type 1 protease biosensor assay using bioluminescence resonance energy transfer. J. Virol. Methods.

[13] Hall M.P., Unch J., Binkowski B.F., Valley M.P., Butler B.L., Wood M.G., Otto P., Zimmerman K., Vidugiris G., Machleidt T. (2012). Engineered luciferase reporter from a deep sea shrimp utilizing a novel imidazopyrazinone substrate. ACS Chem. Biol..

[14] England C.G., Ehlerding E.B., Cai W. (2016). NanoLuc: A Small Luciferase Is Brightening Up the Field of Bioluminescence. Bioconjug. Chem..

[15] Inouye S., Sato J.I., Sahara-Miura Y., Tomabechi Y., Sumida Y., Sekine S.I., Shirouzu M., Hosoya T. (2022). Reverse mutants of the catalytic 19 kDa mutant protein (nanoKAZ/nanoLuc) from Oplophorus luciferase with coelenterazine as preferred substrate. PLoS ONE.

[16] Shaner N.C., Lambert G.G., Chammas A., Ni Y., Cranfill P.J., Baird M.A., Sell B.R., Allen J.R., Day R.N., Israelsson M. (2013). A bright monomeric green fluorescent protein derived from Branchiostoma lanceolatum. Nat. Methods.

[17] den Hamer A., Dierickx P., Arts R., de Vries J., Brunsveld L., Merkx M. (2017). Bright Bioluminescent BRET Sensor Proteins for Measuring Intracellular Caspase Activity. ACS Sens..

[18] Di Antonio V., Palu G., Alvisi G. (2021). Live-Cell Analysis of Human Cytomegalovirus DNA Polymerase Holoenzyme Assembly by Resonance Energy Transfer Methods. Microorganisms.

[19] Messa L., Celegato M., Bertagnin C., Mercorelli B., Alvisi G., Banks L., Palu G., Loregian A. (2021). The Dimeric Form of HPV16 E6 Is Crucial to Drive YAP/TAZ Upregulation through the Targeting of hScrib. Cancers.

[20] Trevisan M., Di Antonio V., Radeghieri A., Palu G., Ghildyal R., Alvisi G. (2018). Molecular Requirements for Self-Interaction of the Respiratory Syncytial Virus Matrix Protein in Living Mammalian Cells. Viruses.

[21] Scaturro P., Trist I.M., Paul D., Kumar A., Acosta E.G., Byrd C.M., Jordan R., Brancale A., Bartenschlager R. (2014). Characterization of the mode of action of a potent dengue virus capsid inhibitor. J. Virol..

[22] Smith K.M., Di Antonio V., Bellucci L., Thomas D.R., Caporuscio F., Ciccarese F., Ghassabian H., Wagstaff K.M., Forwood J.K., Jans D.A. (2018). Contribution of the residue at position 4 within classical nuclear localization signals to modulating interaction with importins and nuclear targeting. Biochim. Biophys. Acta.

[23] Petersen G.F., Pavan S., Ariawan D., Tietz O., Nematollahzadeh S., Sarker S., Forwood J.K., Alvisi G. (2025). Nuclear trafficking of Anelloviridae capsid protein ORF1 reflects modular evolution of subcellular targeting signals. Virus Evol..

[24] Sinigalia E., Alvisi G., Mercorelli B., Coen D.M., Pari G.S., Jans D.A., Ripalti A., Palu G., Loregian A. (2008). Role of homodimerization of human cytomegalovirus DNA polymerase accessory protein UL44 in origin-dependent DNA replication in cells. J. Virol..

[25] Jager S., Cimermancic P., Gulbahce N., Johnson J.R., McGovern K.E., Clarke S.C., Shales M., Mercenne G., Pache L., Li K. (2011). Global landscape of HIV-human protein complexes. Nature.

[26] Cross E.M., Akbari N., Ghassabian H., Hoad M., Pavan S., Ariawan D., Donnelly C.M., Lavezzo E., Petersen G.F., Forwood J.K. (2024). A functional and structural comparative analysis of large tumor antigens reveals evolution of different importin alpha-dependent nuclear localization signals. Protein Sci..

[27] Elbadawy H.M., Mohammed Abdul M.I., Aljuhani N., Vitiello A., Ciccarese F., Shaker M.A., Eltahir H.M., Palu G., Di Antonio V., Ghassabian H. (2020). Generation of Combinatorial Lentiviral Vectors Expressing Multiple Anti-Hepatitis C Virus shRNAs and Their Validation on a Novel HCV Replicon Double Reporter Cell Line. Viruses.

[28] Sinigalia E., Alvisi G., Segre C.V., Mercorelli B., Muratore G., Winkler M., Hsiao H.H., Urlaub H., Ripalti A., Chiocca S. (2012). The human cytomegalovirus DNA polymerase processivity factor UL44 is modified by SUMO in a DNA-dependent manner. PLoS ONE.

[29] Ghassabian H., Falchi F., Timmoneri M., Mercorelli B., Loregian A., Palu G., Alvisi G. (2021). Divide et impera: An In Silico Screening Targeting HCMV ppUL44 Processivity Factor Homodimerization Identifies Small Molecules Inhibiting Viral Replication. Viruses.

[30] Alvisi G., Roth D.M., Camozzi D., Pari G.S., Loregian A., Ripalti A., Jans D.A. (2009). The flexible loop of the human cytomegalovirus DNA polymerase processivity factor ppUL44 is required for efficient DNA binding and replication in cells. J. Virol..

[31] Perales C., Carrasco L., Ventoso I. (2003). Cleavage of eIF4G by HIV-1 protease: Effects on translation. FEBS Lett..

[32] Alvisi G., Manaresi E., Cross E.M., Hoad M., Akbari N., Pavan S., Ariawan D., Bua G., Petersen G.F., Forwood J. (2023). Importin alpha/beta-dependent nuclear transport of human parvovirus B19 nonstructural protein 1 is essential for viral replication. Antivir. Res..

[33] Alvisi G., Manaresi E., Pavan S., Jans D.A., Wagstaff K.M., Gallinella G. (2025). Avermectins Inhibit Replication of Parvovirus B19 by Disrupting the Interaction Between Importin alpha and Non-Structural Protein 1. Viruses.

[34] Ravlo E., Ianevski A., Schjolberg J.O., Solvang V., Dumaru R., Lysvand H., Hankinson J., Vaha-Koskela M., Vainionpaa S., Varhe A. (2025). Synergistic combination of orally available safe-in-man pleconaril, AG7404, and mindeudesivir inhibits enterovirus infections in human cell and organoid cultures. Cell. Mol. Life Sci..

[35] Tripathi A., Chauhan S., Khasa R. (2025). A Comprehensive Review of the Development and Therapeutic Use of Antivirals in Flavivirus Infection. Viruses.

[36] Hilton B.J., Wolkowicz R. (2010). An assay to monitor HIV-1 protease activity for the identification of novel inhibitors in T-cells. PLoS ONE.

[37] Corbel C., Wang Q., Bousserouel H., Hamdi A., Zhang B., Lozach O., Ferandin Y., Tan V.B., Gueritte F., Colas P. (2011). First BRET-based screening assay performed in budding yeast leads to the discovery of CDK5/p25 interaction inhibitors. Biotechnol. J..

[38] Drinovec L., Kubale V., Nøhr Larsen J., Vrecl M. (2012). Mathematical models for quantitative assessment of bioluminescence resonance energy transfer: Application to seven transmembrane receptors oligomerization. Front. Endocrinol..

[39] Mo X.L., Fu H. (2016). BRET: NanoLuc-Based Bioluminescence Resonance Energy Transfer Platform to Monitor Protein-Protein Interactions in Live Cells. Methods Mol. Biol..

[40] Gaber R., Majerle A., Jerala R., Bencina M. (2013). Noninvasive high-throughput single-cell analysis of HIV protease activity using ratiometric flow cytometry. Sensors.

[41] Lv Z., Chu Y., Wang Y. (2015). HIV protease inhibitors: A review of molecular selectivity and toxicity. HIV AIDS Auckl..

[42] Majerova T., Konvalinka J. (2022). Viral proteases as therapeutic targets. Mol. Asp. Med..

[43] Ali R., Ramadurai S., Barry F., Nasheuer H.P. (2018). Optimizing fluorescent protein expression for quantitative fluorescence microscopy and spectroscopy using herpes simplex thymidine kinase promoter sequences. FEBS Open Bio.

