# Supplementary material for: A Highly Sensitive BRET-Based Reporter for Live-Cell Detection of HIV-1 Protease Activity and Inhibitor Screening
Source: Viruses. 2025 Oct 19;17(10):1391. doi: 10.3390/v17101391 (PMC12567724; doi:10.3390/v17101391)
Supplement: Supplementary file 1 [file viruses-17-01391-s001.zip › viruses-3654539-supplementary.pdf]

viruses

MDPI

Article

A Highly Sensitive BRET-Based Reporter for Live-Cell Detection of HIV-1 Protease Activity and Inhibitor Screening

Supplementary Material

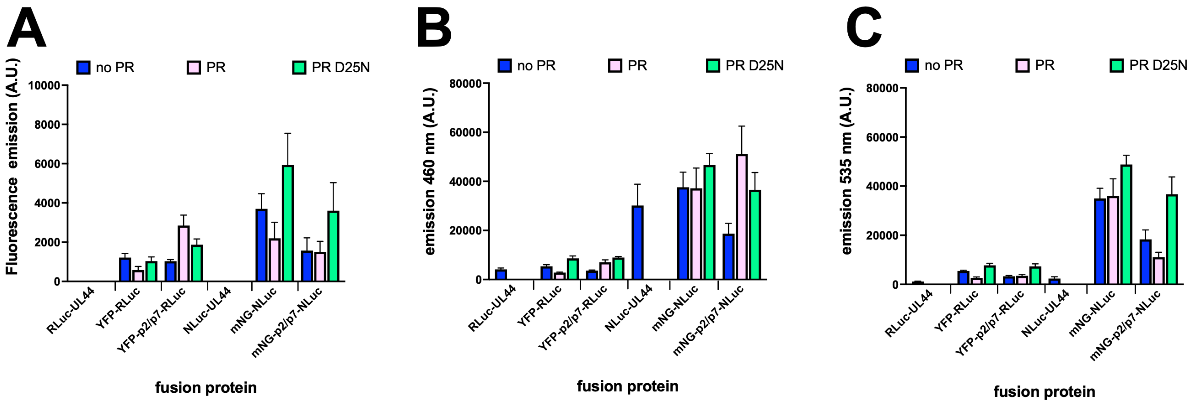

<u>Supplementary Figure S1.</u> **Development of HIV-1 PR BRET cellular biosensors.** HEK293T cells were seeded in 24 well plate and transfected to express the indicated fusion proteins alone or in combination with either HIV-1 PR or its catalytically inactive derivative. 24 hours post transfection cells were transferred to 96 well plates and subjected to BRET assays as described under the Materials and Methos section. The fluorescence emission before addition of H-CTZ (A), as well as the luminometric emission using a 460 $\pm$ 25 nm filter (B), and the luminometric emission using a 535 $\pm$ 25 nm filter (C), 15 minutes after addition of H-CTZ are shown. Data are means $\pm$ standard deviation of the mean, from three independent experiments performed in triplicate.

 https://doi.org/10.3390/v17101391

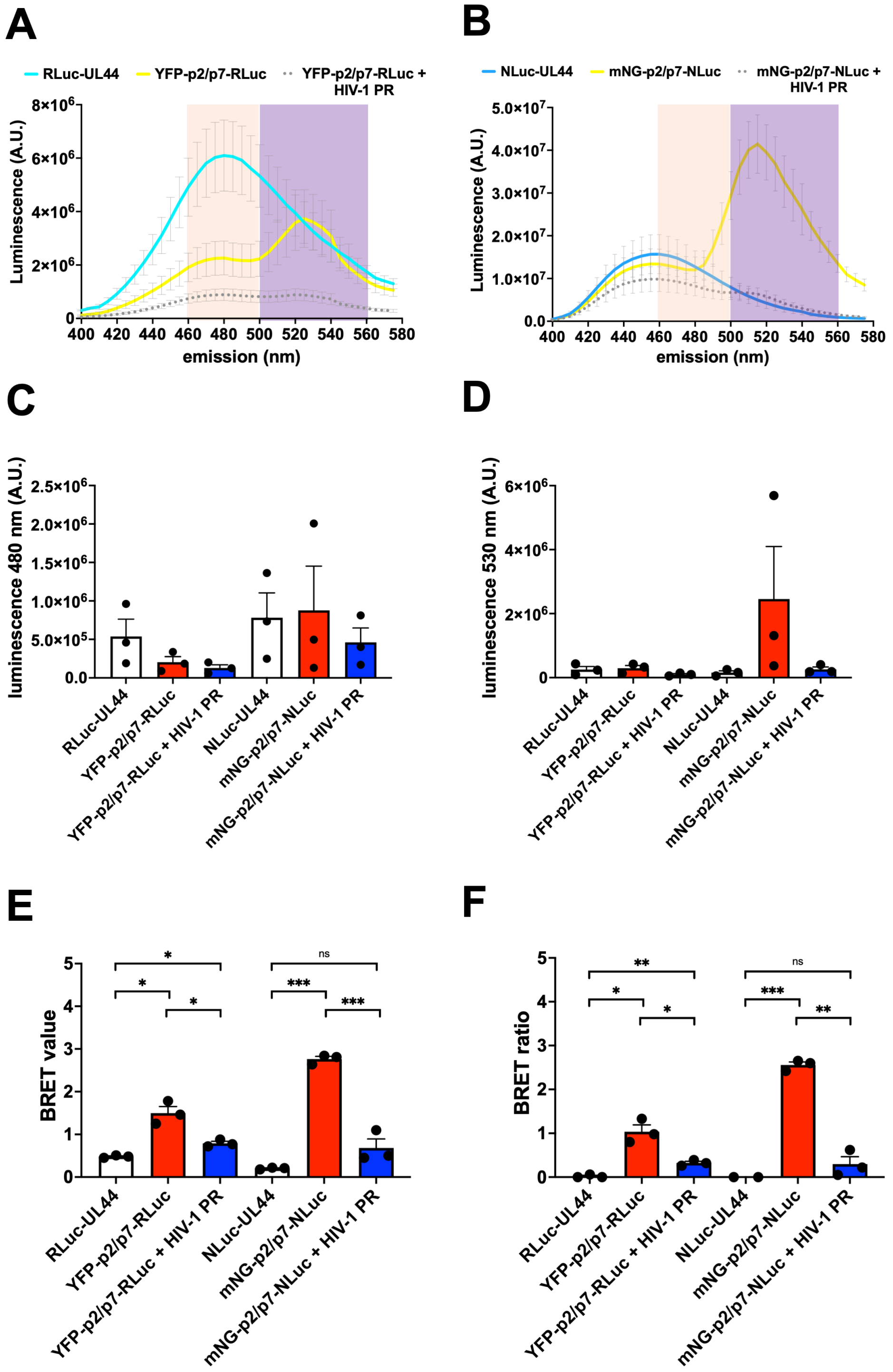

<u>Supplementary Figure S2.</u> Non-normalized emission spectra and BRET measurements corresponding to Figure 3. HEK293T cells were transfected to express the BRET reporters YFP-p2/p7-RLuc and mNG-p2/p7-NLuc in the absence or presence of HIV-1 PR, or the bioluminescent controls RLuc-UL44 and NLuc-UL44. Twenty-four hours post-transfection, cells were processed for luminometric spectral acquisition (A–B) or BRET measurements using 480 ± 20 nm and 530 ± 30 nm band-pass filters (C–F) on a Varioskan LUX plate luminometer, as described in the Materials and Methods. (A–B) Mean light emission spectra of the indicated fusion proteins immediately after addition of h-CTZ, shown as the average of three independent experiments performed in duplicate ± standard error of the mean (SEM). The emission windows corresponding to the 480 ± 20 nm and 530 ± 30 nm filters are boxed in pink and violet, respectively. (C–D) Mean signal intensity detected using the 480 ± 20 nm (C) or 530 ± 30 nm (D) filter for the indicated fusion proteins, shown as the average of three independent experiments performed in duplicate ± SEM. (E) BRET values calculated for the indicated fusion proteins, shown as the average of three independent experiments performed in duplicate ± SEM, together with the results of Brown–Forsythe one-way ANOVA with Welch's correction. (F) BRET ratios calculated for the indicated fusion proteins, shown as the average of three independent experiments performed in duplicate ± SEM, together with the results of Brown–Forsythe one-way ANOVA with Welch's correction.

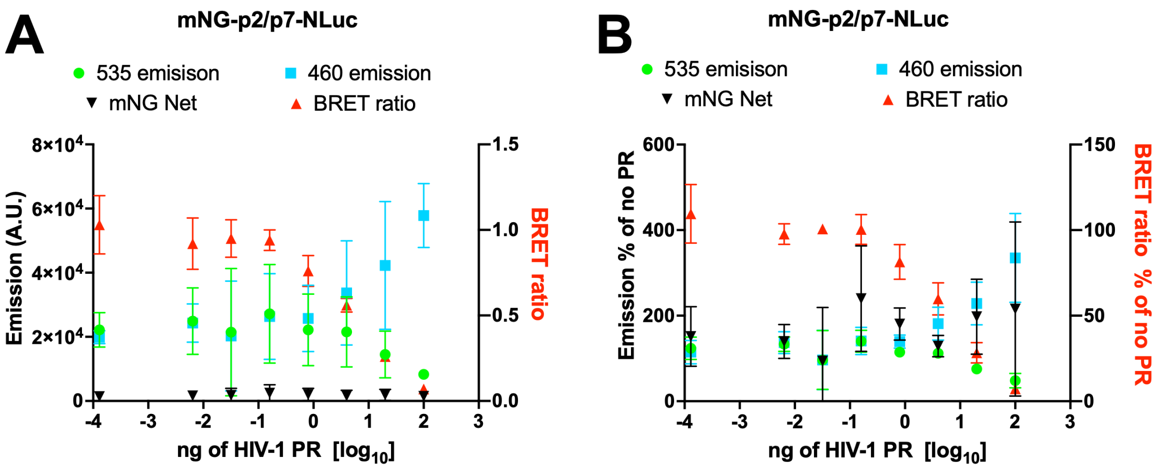

<u>Supplementary Figure S3.</u> **HIV-1 PR dose dependent cleavage of the mNG-p2/p7-NLuc biosensor in living cells.** HEK293T cells were transfected with a fixed amount of mNG-p2/p7-NLuc expression plasmid in the absence or in the presence of increasing amounts of HIV-1 PR expression plasmid**.** Twenty-four hours post transfection cells were processed for BRET measurements as described in the Materials and Methods section. mNG fluorescence emission (black triangles), as well as luminometric emission using a 460 ± 25 nm (cyan squares) and a 535 ± 25 nm (green circles) filter were measured and plotted against the amount of HIV-1 PR expression plasmid used for transfection. Measurements were used to calculate the BRET ratio relative to each condition (red triangles). Data shown are raw values (A) or normalized data expressed as a percentage to those obtained in the absence of HIV-1 PR (B), expressed as means ± standard deviation of the mean relative to three experiments performed in triplicate.

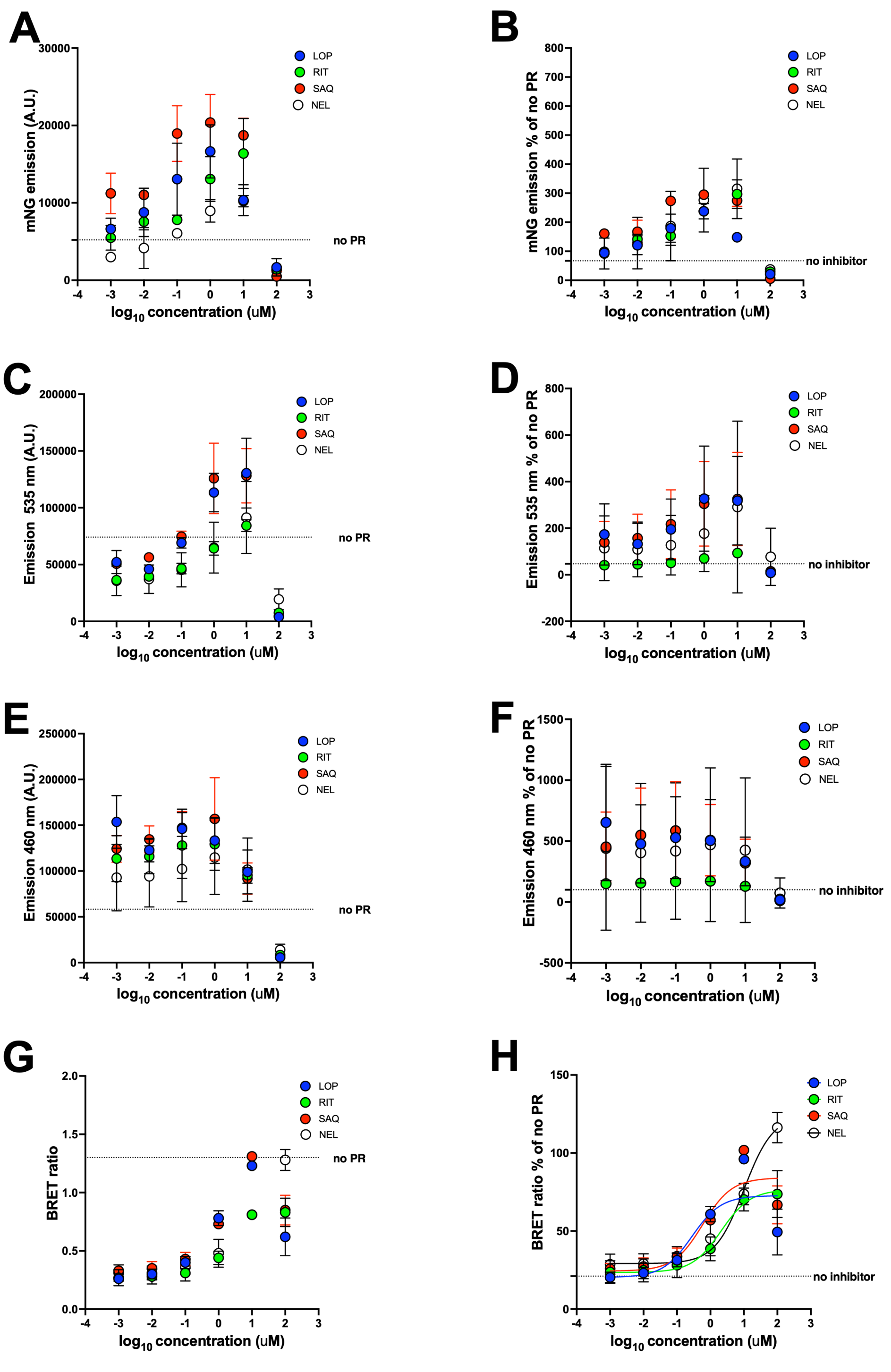

<u>Supplementary Figure S4.</u> Assessment of HIV-1 PR inhibition using mNG-p2/p7-NLuc. HEK293T cells were transfected to express the mNG-p2/p7-NLuc biosensor in the absence or in the presence of HIV-1 PR. Six hours post transfection, media was replaced with fresh media containing either DMSO 1% (v/v) or increasing concentrations of the indicated PR inhibitors. Twenty-four hours post-transfection, cells were transferred to 96 well plates for BRET assays as described in the Materials and Methods section. The fluorescence emission before addition of H-CTZ (A, B), as well as the luminometric emission using a 535 ± 25 nm filter (C, D), the luminometric emission using a 460 ± 25 nm filter (E, F), and the BRET ratio (G, H) 15 minutes after addition of H-CTZ are shown. Data shown are either raw values (A, C, E, G) or normalized data expressed as a percentage to those obtained in the absence of HIV-1 PR (B, D, F, H), expressed as means ± standard deviation of the mean relative to three experiments performed in triplicate, with horizontal dashed lines indicating values obtained in the absence of HIV-1 protease (no PR) or in the presence of HIV-1 protease but in the absence of inhibitors (no inhibitor).
